# Supplementary material for: The Autonomic Nervous System (ANS)-Immune Network in People Living With HIV
Source: Res Sq. 2024 Dec 25:rs.3.rs-5504909. Preprint. [Version 1] doi: 10.21203/rs.3.rs-5504909/v1 (PMC11703338; doi:10.21203/rs.3.rs-5504909/v1)
Supplement: Supplement 1 [file NIHPPRS5504909V1-supplement-1.pdf]

Supplementary Files

This is a list of supplementary files associated with this preprint. Click to download.

- supplementaltables.docx
